# A method for parameter hypothesis testing in nonparametric regression with Fourier series approach

**DOI:** 10.1016/j.mex.2023.102468

**Published:** 2023-10-31

**Authors:** Mustain Ramli, I Nyoman Budiantara, Vita Ratnasari

**Affiliations:** Department of Statistics, Faculty of Science and Data Analytics, Institut Teknologi Sepuluh Nopember, Kampus ITS-Sukolilo, Surabaya 60111, Indonesia

**Keywords:** Nonparametric regression, Fourier series function, Hypothesis testing, Likelihood ratio test, Return on asset, Fourier series function, Likelihood ratio test

## Abstract

Nonparametric regression model with the Fourier series approach was first introduced by Bilodeau in 1994. In the later years, several researchers developed a nonparametric regression model with the Fourier series approach. However, these researches are limited to parameter estimation and there is no research related to parameter hypothesis testing. Parameter hypothesis testing is a statistical method used to test the significance of the parameters. In nonparametric regression model with the Fourier series approach, parameter hypothesis testing is used to determine whether the estimated parameters have significance influence on the model or not. Therefore, the purpose of this research is for parameter hypothesis testing in the nonparametric regression model with the Fourier series approach. The method that we use for hypothesis testing is the LRT method. The LRT method is a method that compares the likelihood functions under the parameter space of the null hypothesis and the hypothesis. By using the LRT method, we obtain the form of the statistical test and its distribution as well as the rejection region of the null hypothesis. To apply the method, we use ROA data from 47 go public banks that are listed on the Indonesia stock exchange in 2020. The highlights of this research are:

• The Fourier series function is assumed as a non-smooth function.
• The form of the statistical test is obtained using the LRT method and is distributed as F distribution.
• The estimated parameters on modelling ROA data have a significant influence on the model.

## Graphical abstract

**Figure:**
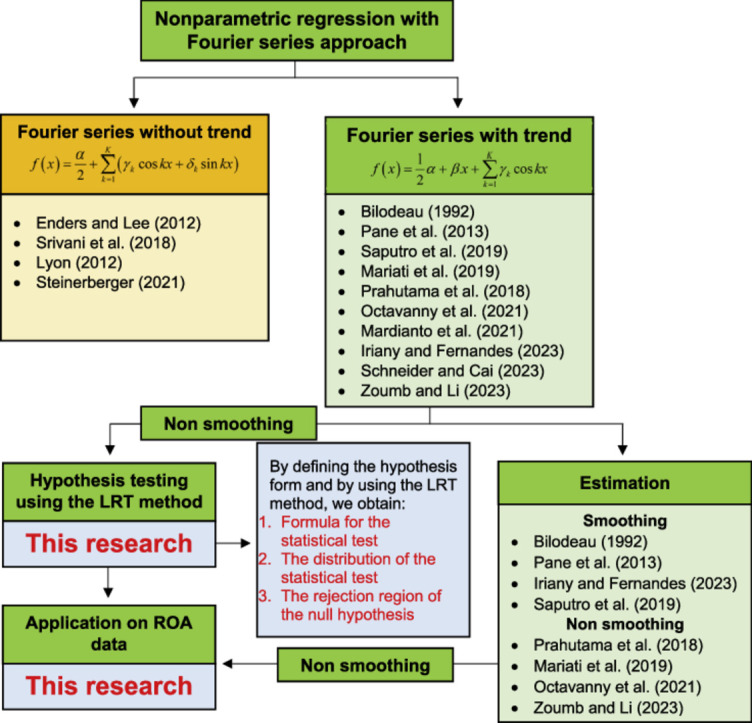

Specifications table

**Table:** 

Subject area:	Mathematics and Statistics
More specific subject area:	Statistics; Nonparametric Regression
Name of your method:	Fourier series function; Likelihood ratio test
Name and reference of original method:	• Fourier series function developed by Bilodeau (1992), M. Bilodeau, Fourier smoother and additive models, The Canadian Journal of Statistics 20 (1992) 257–269. https://doi.org/10.2307/3315313 • Likelihood ratio test in the book of Casella and Berger (2002), G. Casella, R.L. Berger, Statistical Inference, 2nd ed. Pacific Grove: Duxbury Press, 2002.
Resource availability:	Return on asset and its predictor variables data in 2020 of 47 go public banks could be accessed at the Indonesia stock exchange website (https://www.idx.co.id).

**Method details**

The purpose of this research is to develop a method for parameter hypothesis testing in nonparametric regression with the Fourier series approach using the Likelihood Ratio Test (LRT) method and we apply the method to Return on Asset (ROA) data of 47 go public banks on the Indonesia stock exchange in 2020. Based on the hypothesis testing that will be carried out, we will obtain the formula of the statistical test and its distribution as well as the rejection region for the null hypothesis. The method details used in this research are given as follows.

## The model and its estimation

### The nonparametric regression model with Fourier series approach

Regression analysis is part of the statistical method used to model the relationship between predictor and response variables. Suppose $x_{i}$ is the predictor variable and $y_{i}$ is the response variable on the $i^{th}$ observation with i=1,2,...,n, the relationship between $\left(x_{i},y_{i}\right)$ could be expressed as follows.

(1) $$y_{i}=f\left(x_{i}\right)+\varepsilon_{i},$$

where f is the regression curve and $\varepsilon_{i}$ is the error term which we assumed to be normally distributed with mean 0 and the constant variance of $\sigma^{2}$. In regression analysis, there are several approaches for the model (1), namely the parametric regression model and the nonparametric regression model [1]. If we assume f as a known function, then model (1) could be approached using parametric regression. However, if we assume f as an unknown function, then the model (1) could be approached using nonparametric regression. The assumption of f as a known or unknown function could be seen by using a scatterplot [2]. In this research, we are assuming f as an unknown function. Therefore, the model (1) is a nonparametric regression.

Nonparametric regression is a regression approximation which is not bound by the assumption that the shape of the regression curve is known and has flexible properties as the function f could adapt to the nature of the local data. Since f is a nonparametric function, f could be approached using one of the nonparametric estimators. The estimator which could be used to approximate f is the Fourier series function. The Fourier series is a trigonometric polynomial containing cosine and sine functions, which Joseph Fourier first introduced. In 1977, Jong was the first researcher to conduct research related to the Fourier series which discusses the transformation of the Fourier series for smoothing of the density function in the spectral estimator [3]. In the later years, it followed by several researchers, with the form of Fourier series given as of $f\left(x\right)=\frac{\alpha }{2}+\sum_{k=1}^{K}\left(\gamma_{k}\cos\left(kx\right)+\delta_{k}\sin\left(kx\right)\right)$ [4], [5], [6], [7]. However, Bilodeau in 1992 developed the Fourier series function for a smoothing model in nonparametric regression by modifying the function. Bilodeau modifies the function by using the cosine functions only and adds βx as a trend into the Fourier series function [8]. Therefore, the Fourier series function becomes $f\left(x\right)=\frac{1}{2}\alpha +\beta x+\sum_{k=1}^{K}\gamma_{k}\cos\left(kx\right)$. This type of the Fourier series function was developed and used in a nonparametric regression model [see, [9], [10], [11], [12]]. The advantage of using the Fourier series function in nonparametric regression is being able to handle data that has a recurring trend at certain intervals and has a good statistical interpretation.

The nonparametric regression model given in (1) is the nonparametric regression that contains only one predictor variable (univariable model). In this research, we present the number of the predictor variables as p predictor variables (multivariable model). Suppose n is the number of observations and p is the number of the predictor variables, the relationship of the predictor variables and a response variable $\left(x_{i1},x_{i2},...,x_{ip};y_{i}\right)$ assumed to follow the nonparametric regression model as follows.

(2) $$y_{i}=f\left(x_{i1},x_{i2},...,x_{ip}\right)+\varepsilon_{i},\;\;\;i=1,2,...,n,\;\;\;\varepsilon_{i}∼N\left(0,\sigma^{2}\right)$$

If we assume that all the predictor variables are independent or in other words between $x_{1},x_{2},...,x_{p}$ are not correlated, then model (2) could be written in additive model form as follows.

$$y_{i}=f\left(x_{i1},x_{i2},...,x_{ip}\right)+\varepsilon_{i}$$

$$\;=f_{1}\left(x_{i1}\right)+f_{2}\left(x_{i2}\right)+...+f_{p}\left(x_{ip}\right)+\varepsilon_{i}$$

(3) $$\;=\sum_{j=1}^{p}f_{j}\left(x_{ij}\right)+\varepsilon_{i}.$$

Since (2) is the nonparametric regression model then $f_{j}$ are unknown nonparametric regression curves. Let $f_{j}$ are a continuous function, where $f_{j}\in C\left(0,\pi \right)$, then the function of $f_{j}$ could be approximated with the Fourier series function [8].

(4) $$f_{j}\left(x_{ij}\right)=\frac{1}{2}\alpha_{j}+\beta_{j}x_{ij}+\sum_{k=1}^{K}\gamma_{kj}\cos\left(kx_{ij}\right),$$

where $\alpha_{j}$, $\beta_{j}$, and $\gamma_{kj}$ with j=1,2,...p and k=1,2,...K are the parameters in the model and K is the oscillation parameter which represents the number of waves in the cosine function. Therefore, by submitting Eq. (4) into the regression curve of $\sum_{j=1}^{p}f_{j}\left(x_{ij}\right)$ in (3), we simplify have

(5) $$\sum_{j=1}^{p}f_{j}\left(x_{ij}\right)=\sum_{j=1}^{p}\left(\frac{1}{2}\alpha_{j}+\beta_{j}x_{ij}+\sum_{k=1}^{K}\gamma_{kj}\cos\left(kx_{ij}\right)\right).$$

Furthermore, if we describe for j=1,2,...,p and i=1,2,...n, we obtained the Eq. (5) in matrix and vector form as follows.

(6) $$\tilde{f}=X\left(K\right)\tilde{B},$$

where $\tilde{f}=\sum_{j=1}^{p}{\tilde{f}}_{j}$, $X\left(K\right)=\left[\begin{array}{cccc} X_{1}\left(K\right)\;\; & \;\;X_{2}\left(K\right)\;\; & \;\;\cdots \;\; & \;\;X_{p}\left(K\right) \end{array}\right]$, and $\tilde{B}={\left[\begin{array}{cccc} {\tilde{B}}_{1}\;\; & \;\;{\tilde{B}}_{2}\;\; & \;\;\cdots \;\; & \;\;{\tilde{B}}_{p} \end{array}\right]}^{\prime }$, with ${\tilde{f}}_{j}={\left[\begin{array}{cccc} f_{j}\left(x_{1j}\right)\;\; & \;\;f_{j}\left(x_{2j}\right)\;\; & \;\;\cdots \;\; & \;\;f_{j}\left(x_{nj}\right) \end{array}\right]}^{\prime }$,

$$\begin{array}{ccc} & & X_{1}\left(K\right)=\left[\begin{array}{cccccc} \frac{1}{2} & x_{11} & \cos\left(x_{11}\right) & \cos\left(2x_{11}\right) & \cdots & \cos\left(Kx_{11}\right)\\ \frac{1}{2} & x_{21} & \cos\left(x_{21}\right) & \cos\left(2x_{21}\right) & \cdots & \cos\left(Kx_{21}\right)\\ \vdots & \vdots & \vdots & \vdots & \ddots & \vdots \\ \frac{1}{2} & x_{n1} & \cos\left(x_{n1}\right) & \cos\left(2x_{n1}\right) & \cdots & \cos\left(Kx_{n1}\right) \end{array}\right],\;\;X_{2}\left(K\right)=\left[\begin{array}{cccccc} \frac{1}{2} & x_{12} & \cos\left(x_{12}\right) & \cos\left(2x_{12}\right) & \cdots & \cos\left(Kx_{12}\right)\\ \frac{1}{2} & x_{22} & \cos\left(x_{22}\right) & \cos\left(2x_{22}\right) & \cdots & \cos\left(Kx_{22}\right)\\ \vdots & \vdots & \vdots & \vdots & \ddots & \vdots \\ \frac{1}{2} & x_{n2} & \cos\left(x_{n2}\right) & \cos\left(2x_{n2}\right) & \cdots & \cos\left(Kx_{n2}\right) \end{array}\right],...,\\ & & X_{p}\left(K\right)=\left[\begin{array}{cccccc} \frac{1}{2} & x_{1p} & \cos(x_{1p}) & \cos(2x_{1p}) & \cdots & \cos(Kx_{1p})\\ \frac{1}{2} & x_{2p} & \cos(x_{2p}) & \cos(2x_{2p}) & \cdots & \cos(Kx_{2p})\\ \vdots & \vdots & \vdots & \vdots & \ddots & \vdots \\ \frac{1}{2} & x_{np} & \cos(x_{np}) & \cos(2x_{np}) & \cdots & \cos(Kx_{np}) \end{array}\right],\;\begin{array}{c} {\tilde{B}}_{1}={\left[\begin{array}{cccccc} \alpha_{1} & \beta_{1} & \gamma_{11} & \gamma_{21} & \cdots & \gamma_{K1} \end{array}\right]}^{\prime }\\ {\tilde{B}}_{2}={\left[\begin{array}{cccccc} \alpha_{2} & \beta_{2} & \gamma_{12} & \gamma_{22} & \cdots & \gamma_{K2} \end{array}\right]}^{\prime }\\ \;\;\;\vdots \\ {\tilde{B}}_{p}={\left[\begin{array}{cccccc} \alpha_{p} & \beta_{p} & \gamma_{1p} & \gamma_{2p} & \cdots & \gamma_{Kp} \end{array}\right]}^{\prime }. \end{array} \end{array}$$

In general, for i=1,2,...,n, the nonparametric regression model is given in Eq. (3) could be written in matrix and vector form as follows.

(7) $$\tilde{y}=\tilde{f}+\tilde{\varepsilon }=X\left(K\right)\tilde{B}+\tilde{\varepsilon },$$

where $\tilde{y}={\left[\begin{array}{cccc} y_{1} & y_{2} & \cdots & y_{n} \end{array}\right]}^{\prime }$ is the response variable and $\tilde{\varepsilon }={\left[\begin{array}{cccc} \varepsilon_{1} & \varepsilon_{2} & \cdots & \varepsilon_{n} \end{array}\right]}^{\prime }$ is the error term where $\tilde{\varepsilon }∼N\left(\tilde{0},\sigma^{2}I\right)$.

### Parameter estimation

To obtain the estimation of the regression curve of $\tilde{f}$ is equivalent to obtaining the estimation of the parameters. As in many nonparametric regression models, there are many methods to obtain the estimation of the parameters such as the Penalized Least Square (PLS) method if the regression curve of $\tilde{f}$ are assumed to be a smooth function [8,10,13,14]. However, if the regression curve of $\tilde{f}$ is assumed to be only an unknown function and presented as a linear model as of (7), then we could use the Ordinary Least Square (OLS) method that minimizes the sum of the square of the error. By using the optimization of the OLS method, the parameter estimation of $\tilde{B}$ could be obtained as follows [12,[15], [16], [17]].

(8) $$\hat{\tilde{B}}=\arg\min\left\{{\tilde{\varepsilon }}^{\prime }\tilde{\varepsilon }\right\}\underset{\tilde{B}\in R^{p\left(K+2\right)}}{=\arg\min}\left\{{\left(\tilde{y}-X\left(K\right)\tilde{B}\right)}^{\prime }\left(\tilde{y}-X\left(K\right)\tilde{B}\right)\right\}=A\left(K\right)\tilde{y},$$

where

$$\hat{\tilde{B}}={\left[\begin{array}{cccc} \hat{\tilde{B}} & \hat{\tilde{B}} & \cdots & \hat{\tilde{B}} \end{array}\right]}^{\prime }={\left[\begin{array}{cccccccccccccccc} {\hat{\alpha }}_{1} & {\hat{\beta }}_{1} & {\hat{\gamma }}_{11} & \cdots & {\hat{\gamma }}_{K1} & {\hat{\alpha }}_{2} & {\hat{\beta }}_{2} & {\hat{\gamma }}_{12} & \cdots & {\hat{\gamma }}_{K2} & \cdots & {\hat{\alpha }}_{p} & {\hat{\beta }}_{p} & {\hat{\gamma }}_{1p} & \cdots & {\hat{\gamma }}_{Kp} \end{array}\right]}^{\prime },$$

$$\tilde{y}={\left[\begin{array}{cccc} y_{1} & y_{2} & \cdots & y_{n} \end{array}\right]}^{\prime },\;\text{and}\;A\left(K\right)={\left(X^{\prime }\left(K\right)X\left(K\right)\right)}^{-1}X{\left(K\right)}^{\prime }.$$

### Curve estimation and model selection

Based on Eq. (6) and (8), we obtain the estimation curve of the nonparametric regression with the Fourier series approach. Noted $\tilde{f}$ is the regression curve and $\tilde{B}$ is the parameter in the model which we estimated by $\hat{\tilde{B}}$ as of Eq. (8). Therefore, the estimation of the regression curve of $\tilde{f}$ is $\hat{\tilde{f}}$ as follows.

(9) $$\hat{\tilde{f}}=X\left(K\right)\hat{\tilde{B}}=X\left(K\right)A\left(K\right)\tilde{y}=V\left(K\right)\tilde{y},$$

where $V\left(K\right)=X\left(K\right)A\left(K\right)=X\left(K\right){\left(X^{\prime }\left(K\right)X\left(K\right)\right)}^{-1}X{\left(K\right)}^{\prime }$. The estimation of the regression curve (10) is in matrix and vector form. In general, if we make an analogy by Eq. (6) and (4), then the estimation of the regression curve in nonparametric regression with the Fourier series approach could be written as follows.

(10) $${\hat{f}}_{j}\left(x_{ij}\right)=\frac{1}{2}{\hat{\alpha }}_{j}+{\hat{\beta }}_{j}x_{ij}+\sum_{k=1}^{K}{\hat{\gamma }}_{kj}\cos\left(kx_{ij}\right).$$

Furthermore, the parameter estimation of $\hat{\tilde{B}}$ which we obtain using the OLS method in (8) contains an unknown parameter, namely the oscillation parameter of K. In nonparametric regression with the OLS method, there is always one unknown parameter such as knot in the Spline function, bandwidth in the Kernel function, and oscillation parameter in the Fourier series function. Therefore, to obtain the best parameter estimation which fits into the model is the same way as obtaining the optimum number of K in the Fourier series function. The method which could be used to obtain the optimum number of K is the Cross Validation (CV) or the Generalized Cross Validation (GCV) method. The GCV method has been developed by many researchers [see, 18–19] for the Spline function and Bilodeau for the Fourier series function [8]. The GCV formula for choosing the optimum number of the oscillation parameter K is given as follows (the optimum number of K is obtained based on the minimum value of the GCV method):

(11) $$\text{GCV}\left(K\right)=\frac{\mathrm{MSE}\left(K\right)}{{\left(n^{-1}\mathrm{trace}\left[I-V\left(K\right)\right]\right)}^{2}}=\frac{n^{-1}{\tilde{y}}^{T}{\left(I-V\left(K\right)\right)}^{\prime }\left(I-V\left(K\right)\right)\tilde{y}}{{\left(n^{-1}\mathrm{trace}\left[I-V\left(K\right)\right]\right)}^{2}}.$$

## Parameter hypothesis testing using the LRT method

Parameter hypothesis testing plays an important role in modelling and is part of the statistical inference which is essential in regression analysis. Parameter hypothesis testing is used to determine whether the estimated parameters have a significant influence on the model or not. In nonparametric regression model with the Fourier series approach, parameter hypothesis testing has not been carried out previously. Referring to previous researches, this model was used by several researchers for modelling or even for prediction in various fields/data [see, 12,[15], [16], [17]]. However, these researches were only focused on estimation and modelling. Therefore, it is essential to develop a method for parameter hypothesis testing in a nonparametric regression model with the Fourier series approach. One of the methods could be used for parameter hypothesis testing is the LRT method. The LRT method is a method that compares the goodness of fit of two different models (the model under the null hypothesis and the model under the hypothesis). This method is widely used for parameter hypothesis testing in many regressions analysis [see, [20], [21], [22]].

According to Casella and Berger, the LRT method for hypothesis testing is related to Maximum Likelihood Estimation (MLE) [23]. Let $Z_{1},Z_{2},...,Z_{n}$ are random samples from a population with the Probability Density Function (PDF) of f(z|μ), where μ is a parameter (μ may also be a vector), then the likelihood function could be defined as follows.

$$L\left(\mu |z_{1},z_{2},...,z_{n}\right)=L\left(\mu |z\right)=\prod_{i=1}^{n}f(z_{i}|\mu ).$$

Definition 1 Suppose Θ is the parameter space, then the LRT statistic to test $H_{0}:\mu \in \Theta_{0}$ versus $H_{1}:\mu \in \Theta_{0}^{c}$ is given as follows.

(12) $$\Lambda =\frac{\underset{\Theta_{0}}{\sup}L\left(\mu |z\right)}{\underset{\Theta }{\sup}L\left(\mu |z\right)},\;\;\;0<\Lambda \leq 1,$$

where L(μ|z) is the likelihood function with the parameter of μ and the LRT rejected the null hypothesis in the region of $\left\{z_{1},z_{2},...,z_{n}|\Lambda \leq c\right\}$ where c is any constant number with 0≤c≤1. Suppose $\hat{\mu }$ is the parameter estimation under the parameter space of Θ and ${\hat{\mu }}_{0}$ is the estimation parameter under the parameter space of $\Theta_{0}$ which both are obtained by MLE and maximize the likelihood function. Therefore, the LRT in (12) could be written as follows.

(13) $$\Lambda =\frac{L\left({\hat{\mu }}_{0}|z\right)}{L\left(\hat{\mu }|z\right)}=\frac{\max_{\Theta_{0}}L\left(\mu |z\right)}{\max_{\Theta }L\left(\mu |z\right)},\;\;\;0<\Lambda \leq 1.$$

### The hypothesis form and its parameter space

As the central objective of this research is to develop a method for parameter hypothesis testing in nonparametric regression with the Fourier series approach, the initial step involves the formulation of the hypothesis. Suppose the hypothesis form is given as follows.

(14) $$\begin{array}{ccc} & & H_{0}:\alpha_{1}=...=\alpha_{p}=\beta_{1}=...=\beta_{p}=\gamma_{11}=...=\gamma_{1p}=...=\gamma_{K1}=...=\gamma_{Kp}=0\;\text{vs}\\ & & H_{1}:\;\text{at}\;\text{least}\;\text{one}\;\text{of}\;\;\alpha_{j}\neq 0,\beta_{j}\neq 0,\gamma_{kj}\neq 0,\;\text{for}\;\;j=1,2,...,p\;\;\text{and}\;\;k=1,2,...,K. \end{array}$$

The hypothesis given in (14) is a form of hypothesis which tested two different models (a model without parameters and a model containing at least one of the parameters). Mathematically, the hypothesis form in (14) could be written in the following form.

(15) $$H_{0}:E\left(y_{i}|x_{ij}\right)=\sum_{j=1}^{p}f_{j}\left(x_{ij}\right)=0\;vs\;H_{1}:E\left(y_{i}|x_{ij}\right)=\sum_{j=1}^{p}f_{j}\left(x_{ij}\right)\neq 0.$$

Under the assumption of model (2), we know that $\varepsilon_{i}$ are normally distributed with mean 0 and the constant variance $\sigma^{2}$, the PDF of $\varepsilon_{i}∼N\left(0,\sigma^{2}\right)$ is

(16) $$g\left(\varepsilon_{i}\right)=\frac{1}{\sqrt{2\pi \sigma^{2}}}\exp\left(-\frac{{\left(\varepsilon_{i}-0\right)}^{2}}{2\sigma^{2}}\right)=\frac{1}{\sqrt{2\pi \sigma^{2}}}\exp\left(-\frac{\varepsilon_{i}^{2}}{2\sigma^{2}}\right).$$

Based on (3), where $\varepsilon_{i}=y_{i}-\sum_{j=1}^{p}f_{j}\left(x_{ij}\right)$ with $f_{j}$ are Fourier series function, we obtain the likelihood function of (16) as follows.

(17) $$L\left(y_{1},y_{2},...,y_{n}|\sigma^{2}\right)=\prod_{i=1}^{n}\frac{1}{\sqrt{2\pi \sigma^{2}}}\exp\left(-\frac{{\left(y_{i}-\sum_{j=1}^{p}f_{j}\left(x_{ij}\right)\right)}^{2}}{2\sigma^{2}}\right)={\left(2\pi \sigma^{2}\right)}^{-\frac{n}{2}}\exp\left(-\frac{1}{2\sigma^{2}}\sum_{i=1}^{n}{\left(y_{i}-\sum_{j=1}^{p}f_{j}\left(x_{ij}\right)\right)}^{2}\right).$$

Suppose ω is the parameter space under the null hypothesis and Ω is the parameter space under the hypothesis. Based on the hypothesis form in (14) and the likelihood function (17), then we could define the parameter space under the null hypothesis is $H_{0}\left(\omega \right)$ and the parameter space under the hypothesis is H(Ω) as follows.

(18) $$\omega =\left\{\sigma_{\omega }^{2}\right\}\;\text{and}\;\Omega =\left\{\alpha_{1},...,\alpha_{p},\beta_{1},...,\beta_{p},\gamma_{11},...,\gamma_{1p},...,\gamma_{K1},...,\gamma_{Kp},\sigma_{\Omega }^{2}\right\}$$

### The statistical test

Based on Definition 1, the statistical test for testing the hypothesis form given in (14) could be obtained by comparing the maximum likelihood under the parameter space of the null hypothesis (ω) and the parameter space under the hypothesis (Ω) which is given in Theorem 1. However, before presenting Theorem 1, let's first introduce Lemma 1 and Lemma 2. Lemma 1 provides a summary of how to obtain the maximum likelihood under the parameter space of the null hypothesis (ω) and Lemma 2 provides a summary of how to obtain the maximum likelihood under the parameter space of the hypothesis (Ω).

Lemma 1 *Suppose*ω*is the parameter space under the null hypothesis*(18)*then the maximum of the likelihood function*(17)*is*

(19) $$\max_{\omega }L\left(\omega \right)={\left(2\pi {\hat{\sigma }}_{\omega }^{2}\right)}^{-\frac{n}{2}}\exp\left(-\frac{n}{2}\right),$$

*where*${\hat{\sigma }}_{\omega }^{2}=\frac{{\tilde{y}}^{\prime }\tilde{y}}{n}$.

Proof In this case, the parameter space of ω only contains the variance since we define all the parameters under the null hypothesis to be zero value. By the likelihood function (17) and the parameter space of ω (18), we obtain the likelihood function under the parameter space of ω as follows.

(20) $$L\left(\omega \right)={\left(2\pi \sigma_{\omega }^{2}\right)}^{-\frac{n}{2}}\exp\left(-\frac{1}{2\sigma_{\omega }^{2}}\sum_{i=1}^{n}y_{i}^{2}\right)={\left(2\pi \sigma_{\omega }^{2}\right)}^{-\frac{n}{2}}\exp\left(-\frac{{\tilde{y}}^{\prime }\tilde{y}}{2\sigma_{\omega }^{2}}\right),$$

where $\tilde{y}$ is a vector of the response variable. Furthermore, to obtain the maximum of (20) we estimate the parameter of $\sigma_{\omega }^{2}$ by completing $\frac{\partial \ln L(\omega )}{\partial \sigma_{\omega }^{2}}=0$. The natural logarithm of L(ω) in (20) is given as follows.

(21) $$\ln L\left(\omega \right)=\ln\left({\left(2\pi \sigma_{\omega }^{2}\right)}^{-\frac{n}{2}}\exp\left(-\frac{{\tilde{y}}^{\prime }\tilde{y}}{2\sigma_{\omega }^{2}}\right)\right)=-\frac{n}{2}\ln\left(2\pi \right)-\frac{n}{2}\ln\left(\sigma_{\omega }^{2}\right)-\frac{{\tilde{y}}^{\prime }\tilde{y}}{2\sigma_{\omega }^{2}}.$$

The partial derivative of (21) with respect to $\sigma_{\omega }^{2}$ and equalized to zero as follows.

(22) $$\frac{\partial \ln L\left(\omega \right)}{\partial \sigma_{\omega }^{2}}=0-\frac{n}{2\sigma_{\omega }^{2}}-\frac{{\tilde{y}}^{\prime }\tilde{y}}{2\sigma_{\omega }^{4}}=\frac{1}{2\sigma_{\omega }^{2}}\left(n-\frac{{\tilde{y}}^{\prime }\tilde{y}}{\sigma_{\omega }^{2}}\right)=0.$$

Therefore, by (22) we obtain the estimation of $\sigma_{\omega }^{2}$ is ${\hat{\sigma }}_{\omega }^{2}$ as follows.

(23) $${\hat{\sigma }}_{\omega }^{2}=\frac{{\tilde{y}}^{\prime }\tilde{y}}{n}.$$

Since $\sigma_{\omega }^{2}$ is estimated by ${\hat{\sigma }}_{\omega }^{2}$ (23), the maximum of the likelihood function under the parameter space of ω is

$$\max_{\omega }L\left(\omega \right)={\left(2\pi {\hat{\sigma }}_{\omega }^{2}\right)}^{-\frac{n}{2}}\exp\left(-\frac{{\tilde{y}}^{T}\tilde{y}}{2{\hat{\sigma }}_{\omega }^{2}}\right)={\left(2\pi {\hat{\sigma }}_{\omega }^{2}\right)}^{-\frac{n}{2}}\exp\left(-\frac{1}{2}\frac{{\tilde{y}}^{\prime }\tilde{y}}{\frac{{\tilde{y}}^{\prime }\tilde{y}}{n}}\right)={\left(2\pi {\hat{\sigma }}_{\omega }^{2}\right)}^{-\frac{n}{2}}\exp\left(-\frac{n}{2}\right).□$$

Lemma 2 *Suppose*Ω*is the parameter space under the hypothesis*(18)*then the maximum of the likelihood function*(17)*is*

(24) $$\max_{\Omega }L\left(\Omega \right)={\left(2\pi {\hat{\sigma }}_{\Omega }^{2}\right)}^{-\frac{n}{2}}\exp\left(-\frac{n}{2}\right),$$

*where*${\hat{\sigma }}_{\Omega }^{2}=\frac{{\left(\tilde{y}-X\left(K\right){\hat{\tilde{B}}}_{\Omega }\right)}^{\prime }\left(\tilde{y}-X\left(K\right){\hat{\tilde{B}}}_{\Omega }\right)}{n}$*and*${\hat{\tilde{B}}}_{\Omega }={\left(X^{\prime }\left(K\right)X\left(K\right)\right)}^{-1}X^{\prime }\left(K\right)\tilde{y}$.

Proof Noted that the parameter space of Ω is containing all the parameters in the model (the full model). Under the parameter space of Ω, the likelihood function (17) could be defined as follows.

(25) $$L\left(\Omega \right)={\left(2\pi \sigma_{\Omega }^{2}\right)}^{-\frac{n}{2}}\exp\left(-\frac{1}{2\sigma_{\Omega }^{2}}\sum_{i=1}^{n}{\left(y_{i}-\sum_{j=1}^{p}f_{j}\left(x_{ij}\right)\right)}^{2}\right).$$

Since $f_{j}$ are unknown functions and we approximated by the Fourier series function (4), the likelihood function (25) becomes

(26) $$\begin{array}{ccc} L\left(\Omega \right) & = & {\left(2\pi \sigma_{\Omega }^{2}\right)}^{-\frac{n}{2}}\exp\left(-\frac{1}{2\sigma_{\Omega }^{2}}\sum_{i=1}^{n}{\left(y_{i}-\sum_{j=1}^{p}\left(\frac{1}{2}\alpha_{j}+\beta_{j}x_{ij}+\sum_{k=1}^{K}\gamma_{kj}\cos\left(kx_{ij}\right)\right)\right)}^{2}\right)\left[-4pt\right]\\ & = & {\left(2\pi \sigma_{\Omega }^{2}\right)}^{-\frac{n}{2}}\exp\left(-\frac{1}{2\sigma_{\Omega }^{2}}{\left(\tilde{y}-X\left(K\right){\tilde{B}}_{\Omega }\right)}^{\prime }\left(\tilde{y}-X\left(K\right){\tilde{B}}_{\Omega }\right)\right). \end{array}$$

The likelihood function under the parameter space of Ω (26) could be maximized by obtaining the estimation of ${\tilde{B}}_{\Omega }$ and $\sigma_{\Omega }^{2}$. The estimation of ${\tilde{B}}_{\Omega }$ which easily obtained by completing $\frac{\partial \ln L(\Omega )}{\partial {\tilde{B}}_{\Omega }}=0$ as follows.

(27) $$\begin{array}{ccc} & & \frac{\partial \ln L\left(\Omega \right)}{\partial {\tilde{B}}_{\Omega }}=\frac{-\frac{n}{2}\ln\left(2\pi \sigma_{\Omega }^{2}\right)-\frac{1}{2\sigma_{\Omega }^{2}}\left({\tilde{y}}^{\prime }\tilde{y}-2{{\tilde{B}}^{\prime }}_{\Omega }X^{\prime }\left(K\right)\tilde{y}+{{\tilde{B}}^{\prime }}_{\Omega }X^{\prime }\left(K\right)X\left(K\right){\tilde{B}}_{\Omega }\right)}{\partial {\tilde{B}}_{\Omega }}=0\\ & & \Rightarrow -2X^{\prime }\left(K\right)\tilde{y}+2X^{\prime }\left(K\right)X\left(K\right){\tilde{B}}_{\Omega }=0\\ & & \Rightarrow {\hat{\tilde{B}}}_{\Omega }={\left(X^{\prime }\left(K\right)X\left(K\right)\right)}^{-1}X^{\prime }\left(K\right)\tilde{y}. \end{array}$$

The estimation of $\sigma_{\Omega }^{2}$ which is the same way as we obtain $\sigma_{\omega }^{2}$ in Lemma 1 by completing $\frac{\partial \ln L(\Omega )}{\partial \sigma_{\Omega }^{2}}=0$ as follows.

$$\frac{\partial \ln L\left(\Omega \right)}{\partial \sigma_{\Omega }^{2}}=\frac{-\frac{n}{2}\ln\left(2\pi \right)-\frac{n}{2}\ln\left(\sigma_{\Omega }^{2}\right)-\frac{1}{2\sigma_{\Omega }^{2}}{\left(\tilde{y}-X\left(K\right){\tilde{B}}_{\Omega }\right)}^{\prime }\left(\tilde{y}-X\left(K\right){\tilde{B}}_{\Omega }\right)}{\partial \sigma_{\Omega }^{2}}=0$$

$$\Rightarrow -\frac{1}{2\sigma_{\Omega }^{2}}\left(n-\frac{1}{\sigma_{\Omega }^{2}}{\left(\tilde{y}-X\left(K\right){\tilde{B}}_{\Omega }\right)}^{\prime }\left(\tilde{y}-X\left(K\right){\tilde{B}}_{\Omega }\right)\right)=0$$

(28) $$\Rightarrow {\hat{\sigma }}_{\Omega }^{2}=\frac{{\left(\tilde{y}-X\left(K\right){\tilde{B}}_{\Omega }\right)}^{\prime }\left(\tilde{y}-X\left(K\right){\tilde{B}}_{\Omega }\right)}{n}.$$

By giving fixed ${\tilde{B}}_{\Omega }$ or in other words submitting ${\hat{\tilde{B}}}_{\Omega }$ (27) into ${\hat{\sigma }}_{\Omega }^{2}$ (28), we finally obtain the estimation of $\sigma_{\Omega }^{2}$ is ${\hat{\sigma }}_{\Omega }^{2}$ as follows.

(29) $${\hat{\sigma }}_{\Omega }^{2}=\frac{{\left(\tilde{y}-X\left(K\right){\hat{\tilde{B}}}_{\Omega }\right)}^{\prime }\left(\tilde{y}-X\left(K\right){\hat{\tilde{B}}}_{\Omega }\right)}{n}.$$

Therefore, by submitting ${\hat{\tilde{B}}}_{\Omega }$ (27) and ${\hat{\sigma }}_{\Omega }^{2}$ (29) into the likelihood function (26), we obtain the maximum of the likelihood function under the parameter space of Ω as follows.

$$\max_{\Omega }L\left(\Omega \right)={\left(2\pi {\hat{\sigma }}_{\Omega }^{2}\right)}^{-\frac{n}{2}}\exp\left(-\frac{1}{2{\hat{\sigma }}_{\Omega }^{2}}{\left(\tilde{y}-X\left(K\right){\hat{\tilde{B}}}_{\Omega }\right)}^{\prime }\left(\tilde{y}-X\left(K\right){\hat{\tilde{B}}}_{\Omega }\right)\right)$$

$$={\left(2\pi {\hat{\sigma }}_{\Omega }^{2}\right)}^{-\frac{n}{2}}\exp\left(-\frac{1}{2}\frac{{\left(\tilde{y}-X\left(K\right){\hat{\tilde{B}}}_{\Omega }\right)}^{\prime }\left(\tilde{y}-X\left(K\right){\hat{\tilde{B}}}_{\Omega }\right)}{\frac{{\left(\tilde{y}-X\left(K\right){\hat{\tilde{B}}}_{\Omega }\right)}^{\prime }\left(\tilde{y}-X\left(K\right){\hat{\tilde{B}}}_{\Omega }\right)}{n}}\right)={\left(2\pi {\hat{\sigma }}_{\Omega }^{2}\right)}^{-\frac{n}{2}}\exp\left(-\frac{n}{2}\right).□$$

Furthermore, the statistical test for testing the hypothesis form in (14) could be obtained using the LRT method as of Theorem 1.

Theorem 1 *Given the nonparametric regression model*(3)*with*$f_{j}$*approximated by the Fourier series function*(4), *by using the LRT method, the statistical test for testing the hypothesis in*(14)*is*

(30) $$\Lambda^{*}>c^{*},$$

*where*$\Lambda^{*}=\frac{\frac{{\left(X\left(K\right){\hat{\tilde{B}}}_{\Omega }\right)}^{\prime }\tilde{y}}{d_{1}}}{\frac{{\left(\tilde{y}-X\left(K\right){\hat{\tilde{B}}}_{\Omega }\right)}^{\prime }\left(\tilde{y}-X\left(K\right){\hat{\tilde{B}}}_{\Omega }\right)}{d_{2}}}$*with*$d_{1}$*and*$d_{2}$*are given in*Theorem 2*and*$c^{*}=\left(c^{-\frac{2}{n}}-1\right)\frac{d_{2}}{d_{1}}$*with*0≤c≤1.

Proof Since the error in (3) is assumed to be normally distributed with mean 0 and the constant variance $\sigma^{2}$ and given the hypothesis in (14) with ω is the parameter space under the null hypothesis and Ω is the parameter space under the hypothesis. Therefore, by Definition 1 of the LRT in (13) we obtain

(31) $$\Lambda =\frac{\max_{\omega }L\left(\omega \right)}{\max_{\Omega }L\left(\Omega \right)}.$$

By Lemma 1 and Lemma 2, the LRT in (31) becomes

(32) $$\Lambda =\frac{{\left(2\pi {\hat{\sigma }}_{\omega }^{2}\right)}^{-\frac{n}{2}}\exp\left(-\frac{n}{2}\right)}{{\left(2\pi {\hat{\sigma }}_{\Omega }^{2}\right)}^{-\frac{n}{2}}\exp\left(-\frac{n}{2}\right)}=\frac{{\left(2\pi \right)}^{-\frac{n}{2}}{\left({\hat{\sigma }}_{\omega }^{2}\right)}^{-\frac{n}{2}}}{{\left(2\pi \right)}^{-\frac{n}{2}}{\left({\hat{\sigma }}_{\Omega }^{2}\right)}^{-\frac{n}{2}}}={\left(\frac{{\hat{\sigma }}_{\omega }^{2}}{{\hat{\sigma }}_{\Omega }^{2}}\right)}^{-\frac{n}{2}}.$$

Since ${\hat{\sigma }}_{\omega }^{2}$ and ${\hat{\sigma }}_{\Omega }^{2}$ are given in (23) and (29), we obtain the LRT in (32) as follows.

(33) $$\Lambda ={\left(\frac{\frac{{\tilde{y}}^{\prime }\tilde{y}}{n}}{\frac{{\left(\tilde{y}-X\left(K\right){\hat{\tilde{B}}}_{\Omega }\right)}^{\prime }\left(\tilde{y}-X\left(K\right){\hat{\tilde{B}}}_{\Omega }\right)}{n}}\right)}^{-\frac{n}{2}}={\left(\frac{{\tilde{y}}^{\prime }\tilde{y}}{{\left(\tilde{y}-X\left(K\right){\hat{\tilde{B}}}_{\Omega }\right)}^{\prime }\left(\tilde{y}-X\left(K\right){\hat{\tilde{B}}}_{\Omega }\right)}\right)}^{-\frac{n}{2}}.$$

The component of ${\tilde{y}}^{\prime }\tilde{y}$ in (33) could be described as follows.

$$\begin{array}{ccc} {\tilde{y}}^{\prime }\tilde{y} & = & {\left(\tilde{y}-X\left(K\right){\hat{\tilde{B}}}_{\Omega }+X\left(K\right){\hat{\tilde{B}}}_{\Omega }\right)}^{\prime }\left(\tilde{y}-X\left(K\right){\hat{\tilde{B}}}_{\Omega }+X\left(K\right){\hat{\tilde{B}}}_{\Omega }\right)\\ & = & {\tilde{y}}^{\prime }\tilde{y}-{\left(X\left(K\right){\hat{\tilde{B}}}_{\Omega }\right)}^{\prime }\tilde{y}+{\left(X\left(K\right){\hat{\tilde{B}}}_{\Omega }\right)}^{\prime }\tilde{y}-{\tilde{y}}^{\prime }X\left(K\right){\hat{\tilde{B}}}_{\Omega }+{\left(X\left(K\right){\hat{\tilde{B}}}_{\Omega }\right)}^{\prime }X\left(K\right){\hat{\tilde{B}}}_{\Omega }-{\left(X\left(K\right){\hat{\tilde{B}}}_{\Omega }\right)}^{\prime }X\left(K\right){\hat{\tilde{B}}}_{\Omega }+{\tilde{y}}^{\prime }X\left(K\right){\hat{\tilde{B}}}_{\Omega }\\ & = & {\left(\tilde{y}-X\left(K\right){\hat{\tilde{B}}}_{\Omega }\right)}^{\prime }\left(\tilde{y}-X\left(K\right){\hat{\tilde{B}}}_{\Omega }\right)+2{\left(X\left(K\right){\hat{\tilde{B}}}_{\Omega }\right)}^{\prime }\tilde{y}-{\left(X\left(K\right){\hat{\tilde{B}}}_{\Omega }\right)}^{\prime }X\left(K\right){\hat{\tilde{B}}}_{\Omega } \end{array}$$

.

Since ${\hat{\tilde{B}}}_{\Omega }={\left(X^{\prime }\left(K\right)X\left(K\right)\right)}^{-1}X^{\prime }\left(K\right)\tilde{y}$ (27), then ${\left(X\left(K\right){\hat{\tilde{B}}}_{\Omega }\right)}^{\prime }X\left(K\right){\hat{\tilde{B}}}_{\Omega }={\left(X\left(K\right){\hat{\tilde{B}}}_{\Omega }\right)}^{\prime }\tilde{y}$. Thus,

(34) $${\tilde{y}}^{\prime }\tilde{y}={\left(\tilde{y}-X\left(K\right){\hat{\tilde{B}}}_{\Omega }\right)}^{\prime }\left(\tilde{y}-X\left(K\right){\hat{\tilde{B}}}_{\Omega }\right)+{\left(X\left(K\right){\hat{\tilde{B}}}_{\Omega }\right)}^{\prime }\tilde{y}.$$

By submitting ${\tilde{y}}^{\prime }\tilde{y}$ in (34) into (33), we obtain

(35) $$\Lambda ={\left(\frac{{\left(\tilde{y}-X\left(K\right){\hat{\tilde{B}}}_{\Omega }\right)}^{\prime }\left(\tilde{y}-X\left(K\right){\hat{\tilde{B}}}_{\Omega }\right)+{\left(X\left(K\right){\hat{\tilde{B}}}_{\Omega }\right)}^{\prime }\tilde{y}}{{\left(\tilde{y}-X\left(K\right){\hat{\tilde{B}}}_{\Omega }\right)}^{\prime }\left(\tilde{y}-X\left(K\right){\hat{\tilde{B}}}_{\Omega }\right)}\right)}^{-\frac{n}{2}}={\left(1+\frac{{\left(X\left(K\right){\hat{\tilde{B}}}_{\Omega }\right)}^{\prime }\tilde{y}}{{\left(\tilde{y}-X\left(K\right){\hat{\tilde{B}}}_{\Omega }\right)}^{\prime }\left(\tilde{y}-X\left(K\right){\hat{\tilde{B}}}_{\Omega }\right)}\right)}^{-\frac{n}{2}}.$$

Based on Definition 1, the null hypothesis (14) has a rejection region of $\left\{(\tilde{y},X\left(K\right))|\Lambda \leq c\right\}$ where c is any constant number with 0≤c≤1. Therefore, the LRT in (35) for testing the null hypothesis $\left(H_{0}\right)$ against the hypothesis alternative $\left(H_{1}\right)$ in (14) could be simplified as follows.

$${\left(1+\frac{{\left(X\left(K\right){\hat{\tilde{B}}}_{\Omega }\right)}^{\prime }\tilde{y}}{{\left(\tilde{y}-X\left(K\right){\hat{\tilde{B}}}_{\Omega }\right)}^{\prime }\left(\tilde{y}-X\left(K\right){\hat{\tilde{B}}}_{\Omega }\right)}\right)}^{-\frac{n}{2}}\leq c$$

(36) $$\frac{{\left(X\left(K\right){\hat{\tilde{B}}}_{\Omega }\right)}^{\prime }\tilde{y}}{{\left(\tilde{y}-X\left(K\right){\hat{\tilde{B}}}_{\Omega }\right)}^{\prime }\left(\tilde{y}-X\left(K\right){\hat{\tilde{B}}}_{\Omega }\right)}\geq c^{-\frac{2}{n}}-1.$$

Let $d_{1}$ and $d_{2}$ are the degrees of freedom which are given later in Theorem 2. By multiplying $\frac{d_{2}}{d_{1}}$ for both segments in (36), we obtain the statistical test for the hypothesis in (14) as follows.

$$\frac{\frac{{\left(X\left(K\right){\hat{\tilde{B}}}_{\Omega }\right)}^{\prime }\tilde{y}}{d_{1}}}{\frac{{\left(\tilde{y}-X\left(K\right){\hat{\tilde{B}}}_{\Omega }\right)}^{\prime }\left(\tilde{y}-X\left(K\right){\hat{\tilde{B}}}_{\Omega }\right)}{d_{2}}}\geq \left(c^{-\frac{2}{n}}-1\right)\frac{d_{2}}{d_{1}}$$

$$\Lambda^{*}>c^{*}.$$

 □

### The distribution of the statistical test and the rejection region.

The form of the statistical test that we obtain in Theorem 1 is for testing the hypothesis form in (14). To determine whether the null hypothesis presented in (14) is rejected or fails to be rejected by using the statistical test (30), we need to establish the rejection region for the null hypothesis by determining the distribution of the statistical test. The distribution of the statistical test of $\Lambda^{*}$ is given in Theorem 2. However, to support the proof of Theorem 2, it is necessary to simplify the statistical test of $\Lambda^{*}$ as provided in Corollary 1 below.

Corollary 1 *The statistical test of*$\Lambda^{*}$presented inTheorem 1*could be simplified as follows*.

(37) $$\Lambda^{*}=\frac{\frac{{\left(X\left(K\right){\hat{\tilde{B}}}_{\Omega }\right)}^{\prime }\tilde{y}}{d_{1}}}{\frac{{\left(\tilde{y}-X\left(K\right){\hat{\tilde{B}}}_{\Omega }\right)}^{\prime }\left(\tilde{y}-X\left(K\right){\hat{\tilde{B}}}_{\Omega }\right)}{d_{2}}}=\frac{\frac{{\tilde{y}}^{\prime }V\left(K\right)\tilde{y}}{d_{1}}}{\frac{{\tilde{y}}^{\prime }U\left(K\right)\tilde{y}}{d_{2}}},$$

*where*V(K)*is given in*Eq. (10)*and*U(K)=I−V(K).

Proof Noted that the statistical test of $\Lambda^{*}$ is given by Theorem 1. Let $M={\left(X\left(K\right){\hat{\tilde{B}}}_{\Omega }\right)}^{\prime }\tilde{y}$ and $N={\left(\tilde{y}-X\left(K\right){\hat{\tilde{B}}}_{\Omega }\right)}^{\prime }\left(\tilde{y}-X\left(K\right){\hat{\tilde{B}}}_{\Omega }\right)$, since ${\hat{\tilde{B}}}_{\Omega }$ is given in (27) we could simplify M and N as follows.

(38) $$M={\left(X\left(K\right){\hat{\tilde{B}}}_{\Omega }\right)}^{\prime }\tilde{y}={\tilde{y}}^{\prime }X\left(K\right){\left(X^{\prime }\left(K\right)X\left(K\right)\right)}^{-1}X^{\prime }\left(K\right)\tilde{y}={\tilde{y}}^{\prime }V\left(K\right)\tilde{y}.$$

$$N={\left(\tilde{y}-X\left(K\right){\hat{\tilde{B}}}_{\Omega }\right)}^{\prime }\left(\tilde{y}-X\left(K\right){\hat{\tilde{B}}}_{\Omega }\right)={\tilde{y}}^{\prime }\tilde{y}-2{\left(X\left(K\right){\hat{\tilde{B}}}_{\Omega }\right)}^{\prime }\tilde{y}+{\left(X\left(K\right){\hat{\tilde{B}}}_{\Omega }\right)}^{\prime }X\left(K\right){\hat{\tilde{B}}}_{\Omega }.$$

Followed by (34) that ${\left(X\left(K\right){\hat{\tilde{B}}}_{\Omega }\right)}^{\prime }X\left(K\right){\hat{\tilde{B}}}_{\Omega }={\left(X\left(K\right){\hat{\tilde{B}}}_{\Omega }\right)}^{\prime }\tilde{y}$, thus

(39) $$N={\tilde{y}}^{\prime }\tilde{y}-{\tilde{y}}^{\prime }X\left(K\right){\left(X^{\prime }\left(K\right)X\left(K\right)\right)}^{-1}X^{\prime }\left(K\right)\tilde{y}={\tilde{y}}^{\prime }\left(I-X\left(K\right){\left(X^{\prime }\left(K\right)X\left(K\right)\right)}^{-1}X^{\prime }\left(K\right)\right)\tilde{y}={\tilde{y}}^{T}U\left(K\right)\tilde{y}.$$

Based on (38) and (39) the statistical test of $\Lambda^{*}$ simplified becomes

$$\Lambda^{*}=\frac{\frac{{\left(X\left(K\right){\hat{\tilde{B}}}_{\Omega }\right)}^{\prime }\tilde{y}}{d_{1}}}{\frac{{\left(\tilde{y}-X\left(K\right){\hat{\tilde{B}}}_{\Omega }\right)}^{\prime }\left(\tilde{y}-X\left(K\right){\hat{\tilde{B}}}_{\Omega }\right)}{d_{2}}}=\frac{\frac{{\tilde{y}}^{\prime }V\left(K\right)\tilde{y}}{d_{1}}}{\frac{{\tilde{y}}^{\prime }U\left(K\right)\tilde{y}}{d_{2}}}.□$$

Theorem 2 *Let*$\Lambda^{*}$is the statistical test given byTheorem 1*and it has been simplified by*Corollary 1, *then the statistical test of*$\Lambda^{*}$follows the distribution of$F_{\left(d_{1},d_{2}\right)}$*as follows*.

(40) $$\Lambda^{*}=\frac{\frac{{\left(X\left(K\right){\hat{\tilde{B}}}_{\Omega }\right)}^{\prime }\tilde{y}}{d_{1}}}{\frac{{\left(\tilde{y}-X\left(K\right){\hat{\tilde{B}}}_{\Omega }\right)}^{\prime }\left(\tilde{y}-X\left(K\right){\hat{\tilde{B}}}_{\Omega }\right)}{d_{2}}}∼F_{\left(d_{1},d_{2}\right)},$$

*where*$d_{1}=p\left(K+1\right)+1$*and*$d_{2}=n-\left(p(K+1)+1\right)$, *if the following conditions are fulfilled*.

i. *The distribution of*$\frac{{\tilde{y}}^{\prime }V\left(K\right)\tilde{y}}{\sigma^{2}}$*is*$\chi_{\left(d_{1}\right)}^{2}$*where*$d_{1}=p\left(K+1\right)+1$.
ii. *The distribution of*$\frac{{\tilde{y}}^{\prime }U\left(K\right)\tilde{y}}{\sigma^{2}}$*is*$\chi_{\left(d_{2}\right)}^{2}$*where*$d_{2}=n-\left(p(K+1)+1\right)$.
iii. V(K) and U(K) *are independent*.

Proof Let us divide the denominator and numerator of the statistical test of $\Lambda^{*}$ with $\sigma^{2}$ and by Corollary 1 we have $\Lambda^{*}=\frac{\frac{{\tilde{y}}^{\prime }V\left(K\right)\tilde{y}}{\sigma^{2}d_{1}}}{\frac{{\tilde{y}}^{\prime }U\left(K\right)\tilde{y}}{\sigma^{2}d_{2}}}$. We finally have $\frac{{\tilde{y}}^{\prime }V\left(K\right)\tilde{y}}{\sigma^{2}}$ and $\frac{{\tilde{y}}^{\prime }U\left(K\right)\tilde{y}}{\sigma^{2}}$ we will then obtain the distribution.

i. Since $\frac{{\tilde{y}}^{\prime }V\left(K\right)\tilde{y}}{\sigma^{2}}$ is a quadratic form, we easily obtain its distributed $\chi_{\left(d_{1}\right)}^{2}$ by proving V(K) is a symmetric matrix where $V^{\prime }\left(K\right)=V\left(K\right)$ and idempotent matrix where $V^{2}\left(K\right)=V\left(K\right)$.
  (41) $$V^{\prime }\left(K\right)={\left(X\left(K\right){\left(X^{\prime }\left(K\right)X\left(K\right)\right)}^{-1}X^{\prime }\left(K\right)\right)}^{\prime }=X\left(K\right){\left(X^{\prime }\left(K\right)X\left(K\right)\right)}^{-1}X^{\prime }\left(K\right)=V\left(K\right).$$
  (42) $$\begin{array}{ccc} V^{2}\left(K\right) & = & V^{\prime }\left(K\right)V\left(K\right)={\left(X\left(K\right){\left(X{\left(K\right)}^{\prime }X\left(K\right)\right)}^{-1}X{\left(K\right)}^{\prime }\right)}^{\prime }X\left(K\right){\left(X{\left(K\right)}^{\prime }X\left(K\right)\right)}^{-1}X{\left(K\right)}^{\prime }\\ & = & X\left(K\right){\left(X^{\prime }\left(K\right)X\left(K\right)\right)}^{-1}X^{\prime }\left(K\right)X\left(K\right){\left(X^{\prime }\left(K\right)X\left(K\right)\right)}^{-1}X^{\prime }\left(K\right)\\ & = & X\left(K\right){\left(X^{\prime }\left(K\right)X\left(K\right)\right)}^{-1}X^{\prime }\left(K\right)=V\left(K\right). \end{array}$$
  Based on (41) and (42) the matrix V(K) is symmetric and idempotent, thus $\frac{{\tilde{y}}^{\prime }V\left(K\right)\tilde{y}}{\sigma^{2}}∼\chi_{\left(d_{1}\right)}^{2}$. The degree of freedom could be obtained by $d_{1}=trace\left(V(K)\right)$. Since X(K) is a matrix with n rows and p(K+2) columns where the $l^{th}$ column in X(K) are the same where l=h(K+1)+1+h with h=0,1,2,...,p−1 see Eq. (6) for the column elements of X(K), then
  $$d_{1}=trace\left(V(K)\right)=trace\left(X\left(K\right){\left(X^{\prime }\left(K\right)X\left(K\right)\right)}^{-1}X^{\prime }\left(K\right)\right)=p\left(K+1\right)+1.$$
ii. Following by (i) we also easily obtain the distribution of $\frac{{\tilde{y}}^{\prime }U\left(K\right)\tilde{y}}{\sigma^{2}}$ by showing that U(K) is a symmetric matrix where $U^{\prime }\left(K\right)=U\left(K\right)$ and idempotent matrix where $U^{2}\left(K\right)=U\left(K\right)$.
  (43) $$U^{\prime }\left(K\right)={\left(I-V\left(K\right)\right)}^{\prime }=I^{\prime }-V^{\prime }\left(K\right)=I-V\left(K\right)=U\left(K\right).$$
  $$U^{2}\left(K\right)=U^{\prime }\left(K\right)U\left(K\right)={\left(I-V\left(K\right)\right)}^{\prime }\left(I-V\left(K\right)\right)=I-V^{\prime }\left(K\right)-V\left(K\right)+V^{\prime }\left(K\right)V\left(K\right).$$
  By (41) and (42), we know that $V^{\prime }\left(K\right)=V\left(K\right)$ and $V^{\prime }\left(K\right)V\left(K\right)=V\left(K\right)$, we obtain:
  (44) $$U^{2}\left(K\right)=I-V\left(K\right)=U\left(K\right).$$
  Based on (43) and (44) the matrix U(K) is symmetric and idempotent, thus $\frac{{\tilde{y}}^{\prime }U\left(K\right)\tilde{y}}{\sigma^{2}}∼\chi_{\left(d_{2}\right)}^{2}$. Since I is the identity matrix with the dimension of n×n and followed by $d_{1}$ in (i), then we obtain:
  $$d_{2}=trace\left(U(K)\right)=trace\left(I-V(K)\right)=trace\left(I\right)-trace\left(V(K)\right)=n-\left(p(K+1)+1\right).$$
iii. V(K)and U(K) are independent if V(K)U(K)=0, then V(K)U(K)=V(K)(I−V(K)).
  Since $V^{\prime }\left(K\right)=V\left(K\right)$ and $V^{\prime }\left(K\right)V\left(K\right)=V\left(K\right)$, then V(K)V(K)=V(K), thus
  V(K)U(K)=V(K)−V(K)V(K)=V(K)−V(K)=0.
  Based on (i) and (ii) we have proved that $\frac{{\tilde{y}}^{\prime }V\left(K\right)\tilde{y}}{\sigma^{2}}∼\chi_{\left(p(K+1)+1\right)}^{2}$ and $\frac{{\tilde{y}}^{\prime }U\left(K\right)\tilde{y}}{\sigma^{2}}∼\chi_{\left(n-(p(K+1)+1)\right)}^{2}$ as well as (iii) where V(K) and U(K) are independent. Therefore, the statistical test of $\Lambda^{*}$ given in Theorem 2 followed by Corollary 1 is distributed of $F_{\left(d_{1},d_{2}\right)}$ as follows.
  $$\Lambda^{*}=\frac{\frac{{\tilde{y}}^{\prime }V\left(K\right)\tilde{y}}{\sigma^{2}d_{1}}}{\frac{{\tilde{y}}^{\prime }U\left(K\right)\tilde{y}}{\sigma^{2}d_{2}}}=\frac{\frac{{\left(X\left(K\right){\hat{\tilde{B}}}_{\Omega }\right)}^{\prime }\tilde{y}}{d_{1}}}{\frac{{\left(\tilde{y}-X\left(K\right){\hat{\tilde{B}}}_{\Omega }\right)}^{\prime }\left(\tilde{y}-X\left(K\right){\hat{\tilde{B}}}_{\Omega }\right)}{d_{2}}}∼F_{\left(d_{1},d_{2}\right)}.$$
  Since the statistical test given in Theorem 1 has a rejection region of $\left\{\left(\tilde{y},X\left(K\right)\right)|\Lambda^{*}\geq c^{*}\right\}$ and suppose given α is a significance level where 0<α<1. By Theorem 2, since $\Lambda^{*}∼F_{\left(p(K+2),n-p(K+2)\right)}$ and $c^{*}=\left(c^{-\frac{2}{n}}-1\right)\frac{d_{2}}{d_{1}}$. Thus c could be obtained by integrating the PDF of F distribution and equalizing to α which we also easily obtain that $c^{*}=F_{table}=F_{\left(\alpha ,p(K+1)+1,n-(p(K+1)+1)\right)}$. Therefore, the null hypothesis has a rejection region as follows.
  (45) $$\alpha =P\left(\text{rejected}\;H_{0}|H_{0}\;\text{is}\;\text{true}\right)=P\left(\Lambda^{*}\geq c^{*}|\tilde{B}=\tilde{0}\right)=P\left(\Lambda^{*}\geq F_{\left(\alpha ,p\left(K+1\right)+1,n-\left(p\left(K+1\right)+1\right)\right)}|\tilde{B}=\tilde{0}\right).$$
  Based on (45), the null hypothesis will be rejected if $\Lambda^{*}\geq F_{\left(\alpha ,p(K+1)+1,n-(p(K+1)+1)\right)}$ (for given α) or the null hypothesis will be rejected if the probability value is smaller than the significance level ($P(F\geq \Lambda^{*})<\alpha$) where $P\left(F\geq \Lambda^{*}\right)=\int_{\Lambda^{*}}^{\infty }f(F)dF$ with f(F) is the PDF of F distribution. □

## Method validation

### Data source and analysis steps

We use secondary data to apply the method. The data we use is ROA data that we collected from the annual reports of 47 go public banks in 2020. The 47 go public banks are the banks that carry out stock trading on the Indonesia stock exchange, the list of the 47 go public banks could be seen in Table A1 (see Appendix A). We use ROA data as the response variable (y) and 5 predictor variables (x), the detail of the variables are described in Table 1.

**Table 1:** Variable description.

Variable	Notation	Description
Response	y	Return on asset
Predictor	$x_{1}$	Capital adequacy ratio
	$x_{2}$	Non-performing loan
	$x_{3}$	Net interest margin
	$x_{4}$	Operating expenses and operating revenue
	$x_{5}$	Loan to deposit

### Data analysis steps:

1. Create a scatterplot between the response and all the predictor variables.
2. Assume the relationship between the response and all the predictor variables follow the nonparametric regression model with the Fourier series approach.
3. Choose the optimum number of Kwhen the number of K is the same for all the predictor variables by using the GCV method (11).
4. Choose the optimum number of Kwhen the number of K is different for each predictor variable by using the GCV method (11).
5. Create the best model between step 3 and 4 based on the smallest GCV value.
6. Estimate the parameters.
7. Create the hypothesis form for testing the parameters.
8. Calculate the statistic value based on the statistical test and the probability value based on the distribution of the statistical test.
9. Compare the value between the probability value and the significance level of α.
10. Make a decision and conclusion.

In this research, we use the R programming language for the exploration and analysis of ROA data. To streamline the implementation of the method detailed in this research, we have developed a package (syntax), which was created using R-Studio. This package encompasses the data analysis steps described in this research. We have made this package publicly available to facilitate its application to various datasets. The package can be accessed through the following link (https://rpubs.com/Authorsdataanalysis/1104036).

### Application on ROA data

**Fig. 1:**
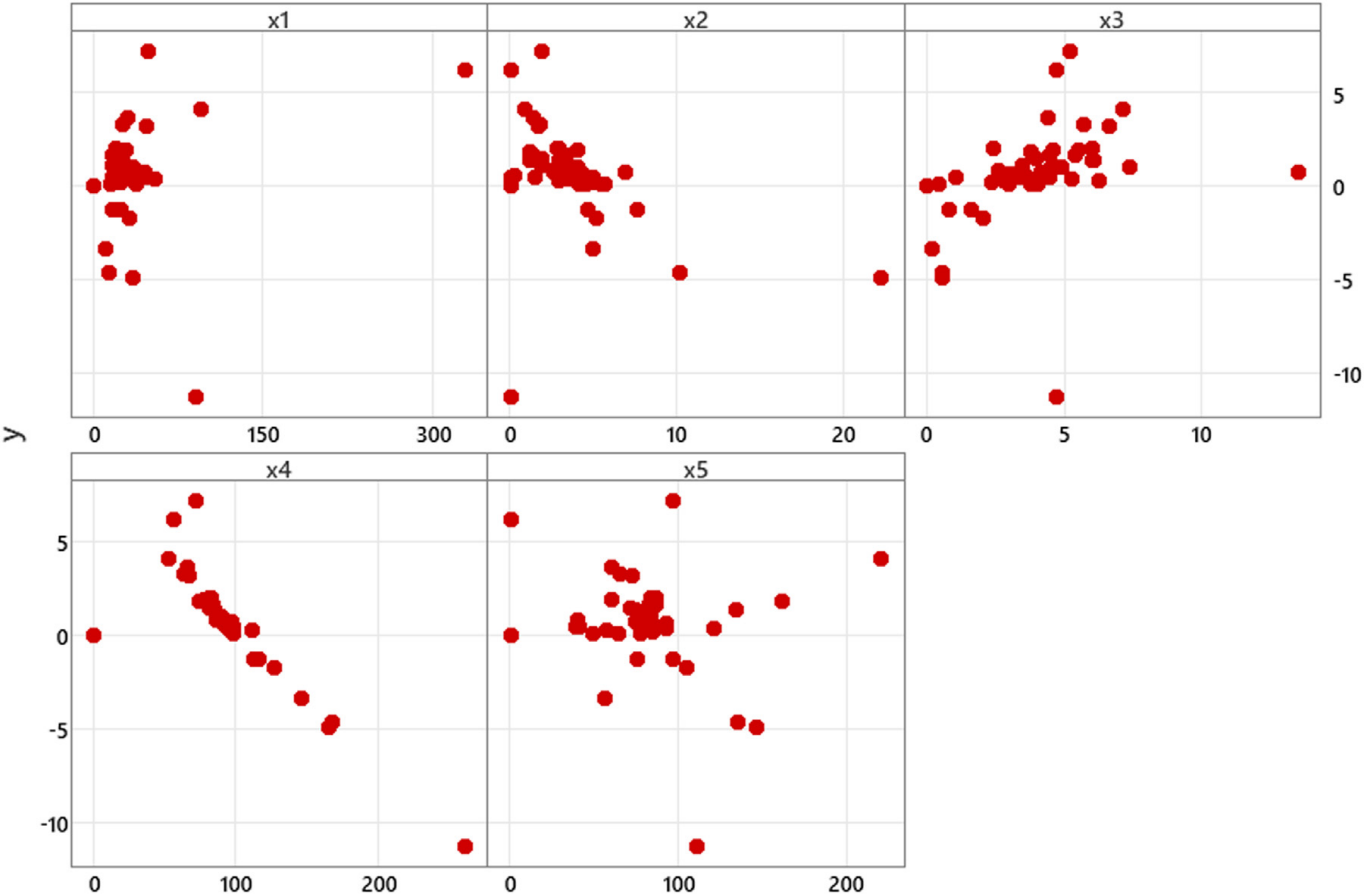
Scatterplots of the response variable and the predictor variables.

We create a scatterplot for each predictor variable versus the response variable to identify that the relationship between the response variable and each predictor variable follow the nonparametric regression model, the scatterplot is given in Fig. 1 as follows.

Based on Fig. 1, we could observe the relationship patterns between the response variable and the predictor variables $x_{1}$, $x_{2}$, $x_{3}$, $x_{5}$ don't exhibit specific patterns, while $x_{4}$ exhibits a tendency toward linearity. However, the relationship patten of $x_{4}$ as linear could not be definitively established without further analysis. To address this, we conducted an analysis for modelling $x_{4}$ using linear parametric regression and we obtained $R^{2}$ of 74.66 % with MSE of 1.898. We also conducted a comparison using nonparametric regression with the Fourier series approach. For the number of K is 1, we obtained $R^{2}$ of 75.52 % with MSE of 1.834. Moreover, we conducted a trial by setting the maximum number of K is 10 and we obtained the optimum number of K is 7 based on the minimum GCV value of 1.803 with $R^{2}$ of 84.26 % and MSE of 1.179. Based on the results of the trial analysis for modelling $x_{4}$ using linear parametric regression and nonparametric regression with the Fourier series approach, we could conclude that, even when initially assessed through scatterplot, $x_{4}$ demonstrates a tendency linearity. However, upon conducted a further analysis, $x_{4}$ is better to be modelled using nonparametric regression with the Fourier series, even when the number of K is 1 or 7 for the maximum number of K is 10 (this could be seen by $R^{2}$ and MSE values). Therefore, in this research, we chose to model the ROA data using nonparametric regression with the Fourier series approach for all the predictor variables.

Since the Fourier series function depends on the number of K, then we use the GCV method to obtain the optimum number of K. In this research, we carried out several trials related to the number of K, including when the number of K is the same for all the predictor variables and the number of K is different for all the predictor variables. In the analysis we conducted, we use the maximum number of K is 5 and we obtained the values of GCV, $R^{2}$, and MSE for the number of K is the same for all the predictor variables in Table 2.

**Table 2:** GCV values for the number of K is the same for all the predictor variables.

K	GCV	$R^{2}$	MSE
1	1.808	85.84 %	1.061
2	1.772	89.71 %	0.771
3	2.033	91.69 %	0.622
4	2.961	92.11 %	0.591
5	2.507	96.12 %	0.291

We obtain the minimum GCV value of 1.772 which means that the optimum number of K is 2. Although the value of $R^{2}$ is highest for the number of K is 5, however the GCV value of 2.507 is highest than the GCV value for the number of K is 2. Therefore, the best estimation model for ROA data by using nonparametric regression with the Fourier series approach is when the number of K is 2 for all the predictor variables. Moreover, we conducted several combinations for the number of K on each predictor variable by taking the maximum number of K is 2, 3, 4, and 5. For the maximum number of K is 2 we have 32 combinations, K is 3 we have 243 combinations, K is 4 we have 1024 combinations, and K is 5 we have 3125 combinations. Based on the results of the analysis, we obtain the minimum of the GCV value as well as the $R^{2}$ and the MSE value for the combination number of K on each predictor variable when the maximum number of K is 2, 3, 4, and 5.

In Table 3 we only show the optimum combination number of K based on the minimum of the GCV value when the maximum number of K is 2, 3, 4, and 5. For example, we take the maximum number of K is 2, then we have 32 combinations for the number of K on all the predictor variables and we obtain the minimum GCV value of 1.537 for the combination number of K is 1 for $x_{1}$, 2 for $x_{2}$, 1 for$x_{3}$, 2 for $x_{4}$, and 1 for $x_{5}$. Based on the possibilities for the combination number of K, we obtain the minimum GCV value of 1.375 when the maximum number of K is 5 with the combination number of K is 5 for $x_{1}$, 2 for $x_{2}$, 3 for$x_{3}$, 2 for $x_{4}$, and 1 for $x_{5}$. Based on the combination number of K on each predictor variable, then we obtain the general form of the nonparametric regression model with the Fourier series approach for ROA data in 2020 as follows.

(46) $$\begin{array}{ccc} y_{i} & = & \frac{1}{2}\alpha_{1}+\beta_{1}x_{i1}+\gamma_{11}\cos\left(x_{i1}\right)+\gamma_{21}\cos\left(2x_{i1}\right)+\gamma_{31}\cos\left(3x_{i1}\right)+\gamma_{41}\cos\left(4x_{i1}\right)+\gamma_{51}\cos\left(5x_{i1}\right)+\frac{1}{2}\alpha_{2}+\beta_{2}x_{i2}+\gamma_{12}\cos\left(x_{i2}\right)\\ & & +\;\gamma_{22}\cos\left(2x_{i2}\right)+\frac{1}{2}{\hat{\alpha }}_{3}+\beta_{3}x_{i3}+\gamma_{13}\cos\left(x_{i3}\right)+\gamma_{23}\cos\left(2x_{i3}\right)+\gamma_{33}\cos\left(3x_{i3}\right)+\frac{1}{2}\alpha_{4}+\beta_{4}x_{i4}+\gamma_{14}\cos\left(x_{i4}\right)\\ & & +\;\gamma_{24}\cos\left(2x_{i4}\right)+\frac{1}{2}\alpha_{5}+\beta_{5}x_{i5}+\gamma_{15}\cos\left(x_{i5}\right)+\varepsilon_{i}. \end{array}$$

**Table 3:** Minimum GCV values for the optimum combinations of K.

Maximum number of K	The optimum combination number of K on each predictor variable					Min GCV	$R^{2}$	MSE
	$x_{1}$	$x_{2}$	$x_{3}$	$x_{4}$	$x_{5}$			
2	K=1	K=2	K=1	K=2	K=1	1.537	89.26 %	0.806
3	K=3	K=2	K=1	K=1	K=1	1.480	90.26 %	0.729
4	K=3	K=2	K=1	K=1	K=1	1.480	90.26 %	0.729
5	K=5	K=2	K=3	K=2	K=1	1.375	93.48 %	0.488

Furthermore, we obtain the estimation of all the parameters in the model (46) as follows.

**Table 4:** Parameter estimations.

Parameters	Estimations	Parameters	Estimations	Parameters	Estimations
$\alpha_{1}$	2.276	$\beta_{2}$	−0.045	$\alpha_{4}$	2.276
$\beta_{1}$	0.020	$\gamma_{12}$	−0.661	$\beta_{4}$	−0.068
$\gamma_{11}$	0.035	$\gamma_{22}$	−0.637	$\gamma_{14}$	−0.414
$\gamma_{21}$	−0.387	$\alpha_{3}$	2.276	$\gamma_{24}$	−0.423
$\gamma_{31}$	−0.352	$\beta_{3}$	0.171	$\alpha_{5}$	2.276
$\gamma_{41}$	−0.137	$\gamma_{13}$	−0.213	$\beta_{5}$	−0.001
$\gamma_{51}$	−0.702	$\gamma_{23}$	0.131	$\gamma_{15}$	−0.265
$\alpha_{2}$	2.276	$\gamma_{33}$	−0.451		

After obtaining the estimation of all the parameters in Table 4, we conducted a parameter hypothesis testing to determine whether all the parameters we estimated have a significant influence on the model (46). Based on the model (46) and the hypothesis form (14) we could define the null hypothesis and the alternative hypothesis for testing the parameters in the model (46) as follows.

(47) $$\begin{array}{ccc} H_{0}:\alpha_{1} & = & \alpha_{2}=\alpha_{3}=\alpha_{4}=\alpha_{5}=\beta_{1}=\beta_{2}=\beta_{3}=\beta_{4}=\beta_{5}=\gamma_{11}=\gamma_{21}=\gamma_{31}=\gamma_{41}=\gamma_{51}=\gamma_{12}=\gamma_{22}=\gamma_{13}=\gamma_{23}=\gamma_{33}\\ & = & \gamma_{14}=\gamma_{24}=\gamma_{15}=0\;vs\;H_{1}:\text{at}\;\text{least}\;\text{one}\;\text{of}\;\text{the}\;\text{parameters}\neq 0 \end{array}$$

Based on the results of the analysis, by using the statistical test in (30) for the number of n is 47, p is 5 and the combination number of K is given in Table 3, we obtain the value of $\Lambda^{*}$ is 22.31. Since $\Lambda^{*}$ is distributed as $F_{\left(d_{1},d_{2}\right)}$ where $d_{1}$ is 19 and $d_{2}$ is 28, by given the significance level of α is 0.05 we obtain $F_{\left(0.05,19,28\right)}$ of 1.97 and the probability value of $P(F\geq \Lambda^{*})$ is 3.82×10^−12^. Since $\Lambda^{*}>F_{\left(0.05,19,28\right)}$ or by the probability value of $P(F\geq \Lambda^{*})<\alpha$, thus we reject the null hypothesis. Therefore, at least one of the parameters is not zero or we could say that all the parameters simultaneously have a significant influence on the model (46).

## Ethics statements

The data we use in this research is secondary data that we collected from the annual report of 47 go public banks on the Indonesia stock exchange in 2020. The data is available on request.

## CRediT authorship contribution statement

**Mustain Ramli:** Conceptualization, Methodology, Software, Writing – original draft, Visualization. **I Nyoman Budiantara:** Conceptualization, Methodology, Writing – review & editing, Validation, Supervision. **Vita Ratnasari:** Conceptualization, Methodology, Writing – review & editing, Validation, Supervision.

## Declaration of Competing Interest

The authors declare that they have no known competing financial interests or personal relationships that could have appeared to influence the work reported in this paper.

## Figures and Tables

**Table A1:** List of 47 go public banks.

No.	Code	Bank name
1	AGRO	PT Bank Raya Indonesia Tbk
2	AGRS	PT Bank IBK Indonesia Tbk
3	AMAR	PT Bank Amar Indonesia Tbk
4	ARTO	PT Bank Jago Tbk
5	BABP	PT Bank MNC Internasional Tbk
6	BACA	PT Bank Capital Indonesia Tbk
7	BANK	PT Bank Aladin Syariah Tbk
8	BBCA	PT Bank Central Asia Tbk
9	BBHI	PT Allo Bank Indonesia Tbk
10	BBKP	PT Bank KB Bukopin Tbk
11	BBMD	PT Bank Mestika Dharma Tbk
12	BBNI	PT Bank Negara Indonesia (Persero) Tbk
13	BBRI	PT Bank Rakyat Indonesia (Persero) Tbk
14	BBSI	PT Krom Bank Indonesia Tbk
15	BBTN	PT Bank Tabungan Negara (Persero) Tbk
16	BBYB	PT Bank Neo Commerce Tbk
17	BCIC	PT Bank Jtrust Indonesia Tbk
18	BDMN	PT Bank Danamon Indonesia Tbk
19	BEKS	PT Bank Pembangunan Daerah Banten Tbk
20	BGTG	PT Bank Ganesha Tbk
21	BINA	PT Bank Ina Perdana Tbk
22	BJBR	PT Bank Pembangunan Daerah Jawa Barat Tbk
23	BJTM	PT Bank Pembangunan Daerah Jawa Timur Tbk
24	BKSW	PT Bank QNB Indonesia Tbk
25	BMAS	PT Bank Maspion Indonesia Tbk
26	BMRI	PT Bank Mandiri (Persero) Tbk
27	BNBA	PT Bank Bumi Arta Tbk
28	BNGA	PT Bank CIMB Niaga Tbk
29	BNII	PT Bank Maybank Indonesia Tbk
30	BNLI	PT Bank Permata Tbk
31	BRIS	PT Bank Syariah Indonesia Tbk
32	BSIM	PT Bank Sinarmas Tbk
33	BSWD	PT Bank of India Indonesia Tbk
34	BTPN	PT Bank BTPN Tbk
35	BTPS	PT Bank BTPN Syariah Tbk
36	BVIC	PT Bank Victoria International Tbk
37	DNAR	PT Bank Oke Indonesia Tbk
38	INPC	PT Bank Artha Graha Internasional Tbk
39	MASB	PT Bank Multiarta Sentosa Tbk
40	MAYA	PT Bank Mayapada Internasional Tbk
41	MCOR	PT Bank China Construction Bank Indonesia Tbk
42	MEGA	PT Bank Mega Tbk
43	NISP	PT Bank OCBC NISP Tbk
44	NOBU	PT Bank Nationalnobu Tbk
45	PNBN	PT Bank Pan Indonesia Tbk
46	PNBS	PT Bank Panin Dubai Syariah Tbk
47	SDRA	PT Bank Woori Saudara Indonesia 1906 Tbk

## Data Availability

Data will be made available on request.

## Appendix A

Table A1
